# flyDetect: An Android Application for Flight Detection

**DOI:** 10.3390/s24186158

**Published:** 2024-09-23

**Authors:** Jonas Reinholdt, Eric Jul, Paulo Ferreira

**Affiliations:** University of Oslo-The Faculty of Mathematics and Natural Sciences-Informatics Department, 0316 Oslo, Norway; jonasre.developer@gmail.com (J.R.); ericbj@ifi.uio.no (E.J.)

**Keywords:** sensors, fly, detection, Android

## Abstract

Over the past years, transport mode recognition has become a large field of research. However, flight as a type of transportation has been mostly overlooked. A system for flight detection might be useful for context-aware applications, but more importantly, it can be used to automatically manage airplane mode on smartphones. Smartphones transmit radio frequency signals which could potentially interfere with aircraft systems, and it is therefore important that devices enable airplane mode to avoid this problem. This paper proposes flyDetect, a method for automatic flight mode detection and an embodiment in the form of an app that demonstrates the viability of the method. Thus, the system uses the accelerometer and barometer in an Android smartphone, can detect the start and end of a flight, and notify other apps or systems on the device when this happens. Our evaluation shows that flyDetect meets the requirements set for the solution, and the results are very promising.

## 1. Introduction

Transport mode recognition has been the topic of multiple scientific papers over the last few years [1,2,3]. Its possible uses are many, e.g., it can be used to find how people get around in a city or be used to control noise canceling in headphones depending on whether the user is walking or on a bus ride. However, research on transport mode recognition has mostly been focused on the most common types of transport such as cars, trams, subways, buses, or walking [4]. Very little research has been published regarding transport mode recognition of flight. This leaves a type of transportation used by millions of people every day undetectable [5].

The output of a flight detection system, i.e., “flying” or “not flying”, could be used to execute any operation. It might prove useful for any context-aware app, but perhaps its best use case is to automatically manage airplane mode on a device. Portable electronic devices (PEDs) that transmit radio frequency signals, such as smartphones, might interfere with aircraft communication and navigation systems and therefore pose a safety hazard [6]. Thus, passengers are required to either power off their PEDs or put them in airplane mode for practically all phases of their flight in order to disable their transmissions. In the event that a passenger does not enable airplane mode (e.g., if a passenger forgets, does not know how because of poor technological familiarity, or if the device is stored in the baggage compartment and therefore not reachable), a flight detection system could automatically enable airplane mode for the passenger. Also, if a passenger forgets to disable airplane mode, the system can take care of this as well.

Thus, the objective of this research was to design and develop a method for automatic flight mode detection and an embodiment in the form of an app that demonstrates the viability of the method. In addition, it should be able to notify other apps or systems on the device when a flight is detected. The requirements for the solution were the following: (i) the app can detect flight when it occurs at least 95% of the time, with a maximum false negative rate of 5%, and no false positives; (ii) the detection is carried out in near real time locally on the device using onboard sensors, independent of external sources of information; (iii) the app should not make use of privacy-delicate sensors such as the microphone; (iv) it is energy-efficient; (v) it does not require user interaction to function; and (vi) it runs on Android.

There have been a few solutions suggested for flight detection and automatic airplane mode [7,8,9]. However, most of them are either unsuitable for use in smartphones or do not solve the problem in a way that is satisfactory. For instance, one proposed solution suggests using the microphone to detect flight [7]; this may raise privacy concerns for users because they can see when the microphone is active (this feature was introduced in Android 12 [10]). Furthermore, this solution was designed for notebooks (mobile personal computers, similar to laptops but smaller) while the focus of this paper is on smartphones. Another proposed solution suggests using airplane WiFi and having the crew manually control when airplane mode should be enabled or disabled [8]. This does not meet the requirement of performing the flight detection locally and independent of external sources of information, since the detection suggested is performed by a human instead of an app. Additionally, the solution will not work if the aircraft does not have onboard WiFi. A third solution is based on accelerometer data and designed for package trackers [9]. This solution enables airplane mode when vibrations reach a predetermined threshold, but this also makes it unfit for smartphones because it would detect flying if a user was walking with the device in their pocket.

It is possible to use GPS to determine when a user is at an airport; however, this approach is labor-intensive as it requires specifying location boundaries for all airports in order to trigger the system in the correct areas. In addition, GPS signal reception inside a aircraft cabin is hampered by the hull, and is in general poor, even misleading, e.g., when signals are reflected off of buildings and received through an aircraft window. In general, a GPS device must be close to a window to receive a reliable signal and even then, the device can only see half the available satellites. Finally, it would not be energy-efficient since the GPS is a high-power-consumption sensor [11], and it would cause false positives for the ground crew working outside the aircraft. Because of the challenges of detecting when the user is in an aircraft, our system detects the start of a flight as the aircraft takes off, and likewise, the end of a flight is detected as the aircraft lands. Thus, in this work, we attempt to create a flight detection system for smartphones.

When developing a flight detection system there are a few challenges to overcome, mainly in the areas of energy and accuracy of the detection. For an app to detect an activity using sensor data (flying in this case) the app must be running and sampling sensor data at the time the activity occurs. This means that the app must be running at all times.

**Figure 1:**
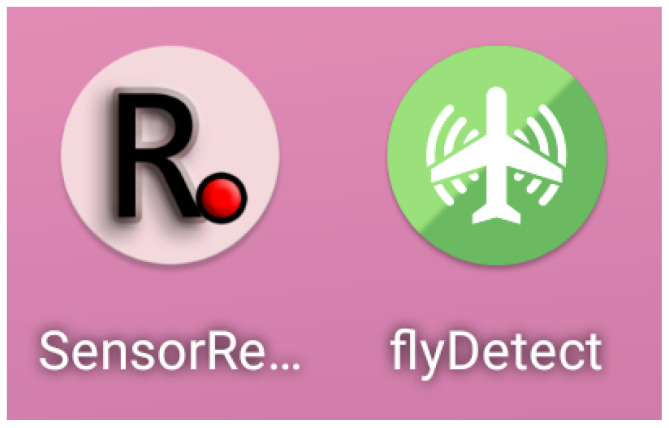
sensorRecord and flyDetect.

This paper includes two applications: sensorRecord and flyDetect (their logos are visible in Figure 1). The sensorRecord app is an Android application that is used extensively throughout the data collection process. Its primary function is to record data from real flights, from the accelerometer and barometer of the device it is running on, as well as to facilitate the labeling of specific events (e.g., takeoff, landing, etc.). The app was also used for recording other activities that were later used to design the algorithm for flyDetect and to evaluate it.

To satisfy the objective and requirements defined above, we propose flyDetect. flyDetect is an Android application that, using only built-in sensors, can detect when a flight starts and when it ends. It achieves this by continuously collecting acceleration data from the accelerometer, which it analyzes at intervals to detect the substantial acceleration that the device experiences during takeoff. Once the app detects a takeoff, it broadcasts to the rest of the device that it is currently flying. At the same time, it stops collecting data from the accelerometer and instead collects pressure data from the barometer. The pressure data are, like acceleration data, analyzed at intervals but this time used to detect landing. To detect landing, the app looks for an increase in pressure that, in the data collected, is observed before landing in all recorded flights. Once landing is detected, the app broadcasts to the rest of the device that it is no longer flying. The app then returns to the state it was in before takeoff was detected, collecting acceleration data and analyzing them at intervals.

The main contribution of this research is a method for automatic flight mode detection and an embodiment in the form of an app that demonstrates the viability of the method. This app detects the beginning and end of a flight in near real time using sensor data and achieves high accuracy with low power usage. To the best of our knowledge, this is the first system developed for smartphones that is capable of this without being dependent on external sources of information or using privacy-delicate sensors (like microphones).

The paper is organized as follows. The next section presents some related work, while Section 3 gives a very short overview of the sensorRecord app. Then, we focus on the algorithm of flyDetect in Section 4. We shortly present how the system was implemented in Section 5. Section 6 and Section 7 present the evaluation results and some conclusions, respectively.

## 2. Related Work

In this section, we present the most relevant related work divided into four categories: (i) automatic airplane mode using microphone, (ii) managing airplane mode with Firebase messaging, (iii) human activity recognition using machine learning, and (iv) flight detection using the accelerometer.

### 2.1. Automatic Airplane Mode Using Microphone

A technical paper from HP Inc. [7] discusses a method of automatically setting airplane mode in notebooks using the built-in microphone to continuously listen to background noise. It describes the sound inside an aircraft cabin as being close to white noise, with take-off and landing being louder than the cruise phase. Furthermore, it states that the noise in the cruise phase is at least 85 dB in commercial planes, while in private jets the noise is at about 50 dB. The algorithm therefore checks if the amplitude is at least 50 dB, and if it is, then a score is incremented. Similarly, if the amplitude is not 50 dB then the score is decremented. This is repeated every 30 s, and if the score surpasses five, the user is flying. If the score later becomes lower than three, then the user is not flying.

Although the goal of that work is similar to the objective of this paper (defined in Section 1), it is inherently different since it targets notebooks instead of smartphones. As of Android 12, users are made aware when either their camera or microphone is in use [10]. This makes that solution problematic since even though it might not store or upload any data, the user will likely be uncomfortable seeing that the microphone is always active. Thus, the user will be uncomfortable for privacy reasons. Because of this, the user might decide to uninstall the app.

There is no mention of test results in that paper. Thus, it is unclear how well this solution performs in practice.

### 2.2. Managing Airplane Mode with Firebase Messaging

A recent conference paper by Sengar and Rawat [8] looks into solutions to disable all possible interference from smartphones. It mentions solutions like mobile-phone jamming and onboard cellular picocells, but also a system for automatic enabling of airplane mode. The system consists of passengers with an app installed in their smartphones, all of them connected to the local airplane WiFi, and cabin crew manually controlling when airplane mode should be enabled (this is a bit unclear in the paper). When airplane mode should be enabled, a Firebase message is sent to all apps connected to the local WiFi. The app then displays a notification to the passenger, requesting that they enable airplane mode. Similar notifications are displayed every 60 s. If airplane mode is still not enabled, then the system automatically enables it. When airplane mode is to be disabled, a new Firebase message is sent to all apps (after enabling local WiFi) so that a new notification is displayed to the passenger.

The solution proposed by their paper does not solve the problem of detecting flight, since this is performed manually by the cabin crew. The paper describes the activation of airplane mode in the system as an “external system trigger”, in the sense that the activation signal comes from an external source and not the smartphone itself.

### 2.3. Human Activity Recognition Using Machine Learning

Human activity recognition (HAR) is a process with the goal of classifying human actions, typically using sensor data. A recent paper by Straczkiewicz et al. [3] reviewed and compared methods for HAR from 108 different papers. The paper found that most approaches were divided into four stages:

1. Data acquisition: the process of data collection, for instance, when the app is collecting raw sensor data;
2. Data preprocessing: the process of cleaning up and transforming the results of the data acquisition;
3. Feature extraction: a process that helps discover unique patterns in the data;
4. Activity classification is the process of assigning an activity to the data received. This is typically performed by a machine learning algorithm.

The accelerometer seems to be the most widely used sensor for HAR—the authors found that 97.2% of the reviewed papers proposed solutions using it, with a lower prevalence of sensors such as gyroscope = 49.1%, magnetometer = 25.9%, GPS = 13.0%, and other sensors = 19.4%.

The rotational motion data provided by the gyroscope could be useful to detect the change in pitch when an airplane is taking off, but for this to work the phone must be in a fixed position relative to the aircraft. In practice, this is not the case, since passengers might hold the phone in their hands and rotate it on different axes. Similarly, the magnetometer may provide the smartphone with data about its orientation in space, but this information is worthless for detecting flight. Although numerous papers follow the same approach, machine learning is impractical because of its dependence on huge volumes of data.

### 2.4. Flight Detection Using Accelerometer

A paper from 2013 by Tawk et al. [9] proposed a solution to automatically disable all wireless functionality when a flight is detected. This is similar to the functionality of airplane mode in smartphones today; however, that solution was designed for a package tracking system that allowed customers to track their package anywhere in the world, almost in real time. The approach relied exclusively on data from an accelerometer and is focused on low power usage.

Because of the situations this solution would be deployed in, i.e., being left unattended in the cargo bay of an aircraft, it was of utmost importance that flight was always detected. As a result of this, the solution did not distinguish between different types of transport but would instead always disable wireless functionality if movement was detected.

The solution was developed by first gathering sensor data when the device was laying still and not moving, and then calculating the moving variance. Using this, the researchers could find out how noisy the accelerometer data were. After this, sensor data were gathered during a flight, and the moving variance was once again calculated. The collected data showed a noticeable difference when engines were running compared to when they were not, and both takeoff and landing created significant spikes. With this, it was possible to determine a threshold that, if exceeded by the moving variance of the sensor data, would infer that the device was in an aircraft with its engines running.

For its purpose, this solution was adequate in all ways; however, it is not ideal for smartphones. Because of the importance of detecting flight when it happens (i.e., in real time), the solution was very sensitive to movement. Therefore, had it been developed for smartphones, it would likely enable airplane mode whenever the user is walking or using other means of transport. Additionally, smartphones are often in the hands or pockets of a passenger when flying, and this is likely to have a shock-absorbing effect. Because of this, training a machine learning model on accelerometer data from engine vibration probably would not provide good accuracy.

## 3. Overview of sensorRecord

**Figure 2:**
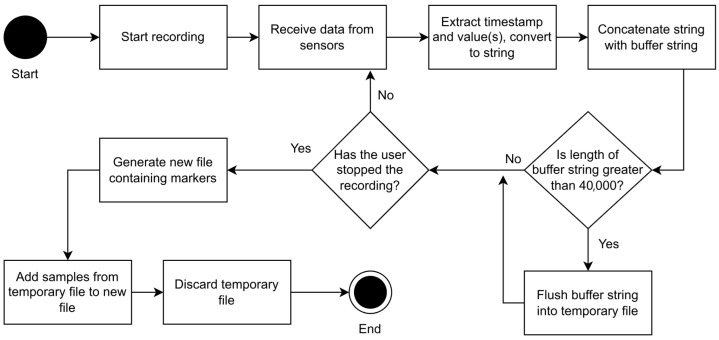
Flowchart of the data collection app sensorRecord.

We now provide a very short overview of the sensorRecord app (see Figure 2) so that the application running in the smartphone can be easily used.

### 3.1. Data Collection App

Designing and tweaking a system for flight detection requires some sensor data from real flights. To acquire the necessary data, we developed a data collection app called sensorRecord. The primary purpose of the app was to record data from the accelerometer and barometer; it allowed the user to label specific events as they occurred so that the different phases of a flight were easily identifiable when the data were examined later.

### 3.2. User Instructions

To achieve a consistent structure in the collected data, for each flight, the user (data collector) was requested to follow these instructions (which were also included in the app interface):

1. Start the recording once seated in the aircraft;
2. Add a marker for each of these events: **pushback** (when the aircraft leaves the gate); **taxi** (when the aircraft starts moving using its own engines, which typically occurs after the aircraft has been stationary for a short while after pushback); **takeoff** (when the aircraft is on the runway and you hear the engines increase power, i.e., not when the aircraft lifts off, but when it starts accelerating); **landing** (the second the aircraft touches the ground again); **park** (when the aircraft stops at the gate);
3. Stop the recording once outside the aircraft.

## 4. Algorithm

**Figure 3:**
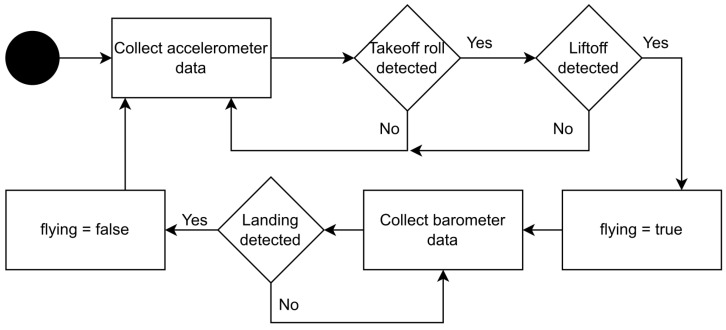
Simplified overview of the entire flyDetect algorithm.

The proposed algorithm of flyDetect (see Figure 3) utilizes two built-in sensors in the device: the accelerometer and the barometer, which provide acceleration and pressure data, respectively. Only one sensor is used at a time depending on the state of the algorithm, of which there are two:

- **“Not flying”**: the algorithm believes that the user is not flying and is therefore continuously monitoring data from the accelerometer. The state is switched to “flying”, if a takeoff is detected.
- **“Flying”**: the algorithm believes that the user is flying and is therefore continuously monitoring data from the barometer to attempt to detect landing. The state is switched to “not flying” if a landing is detected.

Switching the state for each detection (can also be forced by the user) means that the algorithm can run in an infinite cycle until interrupted.

The sensor data utilized in takeoff and landing detection require a timestamp of when the measurement was performed and the value measured by the sensor. The timestamp is required because the algorithm is based on observing changes in sensor data over time. The data are analyzed at intervals, therefore the algorithm schedules a time when this happens and wait until a sample with the same or a later timestamp arrives.

All sensor data that the algorithm receives are stored in a circular buffer that is shared among both sensors (i.e., the accelerometer and the barometer) since only one of them is active at a time. Once the scheduled time for analysis is reached (which is typically every 60 s), the algorithm retrieves the latest data and attempts to detect takeoff or landing. The quantity of data retrieved depends on whether it originated from the accelerometer or the barometer as the sampling frequency of the accelerometer is higher than that of the barometer.

For takeoff detection, the algorithm looks for an increase in acceleration that occurs when an aircraft is on the runway gaining speed. If this is detected, the algorithm then looks for a more intense but shorter duration increase in acceleration that occurs as the aircraft becomes airborne. For this event, the acceleration increase has to be within a specified range for a specified duration to be considered valid (more details later).

For landing detection, the algorithm seeks an increase in pressure that occurs when landing in an aircraft with pressurized cabins. As with takeoff detection, this increase is required to be within a specified range and not longer than a specified duration. If the conditions are met, the algorithm determines that the user has landed. Figure 3 illustrates a simplified overview of the entire algorithm.

### 4.1. Takeoff Detection

**Figure 4:**
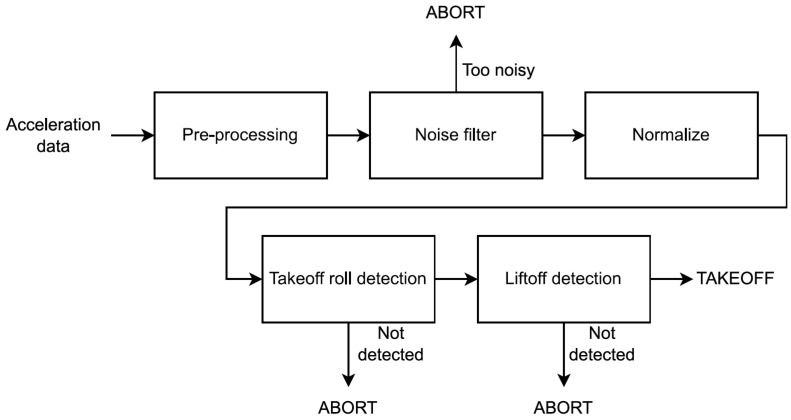
Overview of takeoff and liftoff detection algorithm.

The takeoff detection algorithm relies solely on acceleration data (see Figure 4). Although these data are provided in three dimensions (x, y, and z-axis), we instead chose to calculate and use its full magnitude. With this approach, using a gyroscope to keep track of orientation becomes unnecessary, thus the additional power drain from using a gyroscope is avoided. The algorithm takes advantage of a unique two-stage pattern seen in acceleration during takeoff; it is increased when the aircraft is rolling down the runway (takeoff roll) and further increased when the aircraft becomes airborne (liftoff). This is detected by calculating the moving average of the acceleration, and then identifying when these data are in the correct range for a specified time duration. This detection method is applied to both takeoff roll and liftoff but with separate values for the range and time duration parameters.

#### 4.1.1. Preprocessing

The flyDetect algorithm starts by obtaining the last 60 s of acceleration data (stored in a circular buffer). In this stage, the algorithm calculates the moving average and the moving variance of these data, both with a window size of 10 s.

Once a data point has been calculated, the window slides forward by one sample and the calculation is repeated for the next data point. However, as the window approaches the end of the data from which the moving average/variance is calculated, eventually, there are not enough seconds (10 s, to be more precise) of data left. Thus, when there is an insufficient quantity of data left to fill the window, the calculation is stopped. As a consequence, the duration of the moving average/variance is always a few seconds shorter than the original data it was derived from.

The window size of 10 s for moving average was chosen to sufficiently smooth out the significant noise that is present in acceleration data. This is crucial for the accuracy of the algorithm since it relies on acceleration being within a certain range for a specified minimum time to detect takeoff-related events (more details later), and a single data point outside of the specified range would leave the event undetected. The same window size is used for moving variance, which makes the variance less sensitive to small changes in acceleration and makes it easier to identify trends in the data. A bigger window would still work fine; however, it would truncate the data further, thus increasing the delay in detection time.

The decision to aim for a total of 60 s of acceleration data to analyze was made due to the importance of low latency in detection (as mentioned in Section 1).

#### 4.1.2. ”Noise Filter”

**Figure 5:**
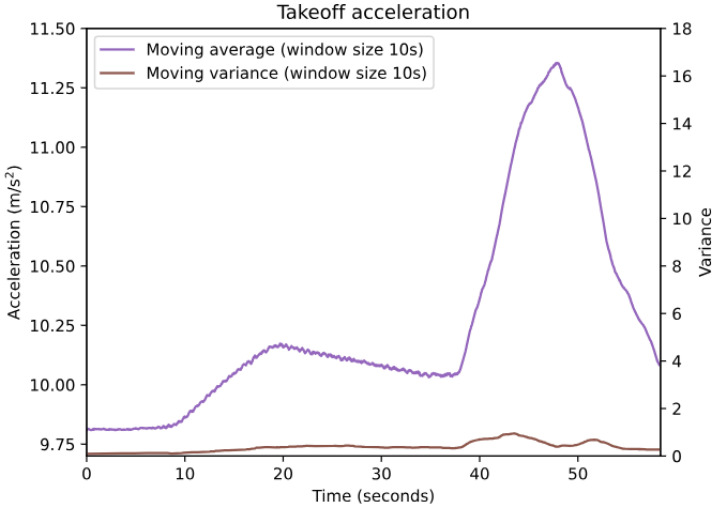
Takeoff acceleration and variance.

**Figure 6:**
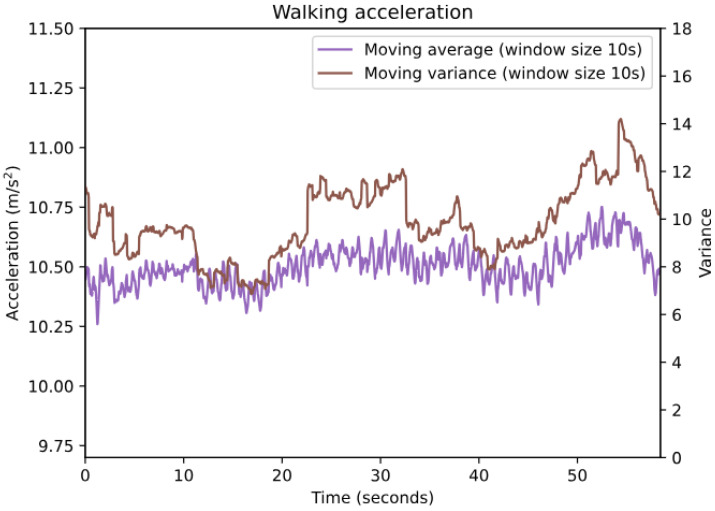
Walking acceleration and variance.

The next step of the algorithm is to check if the acceleration is “calm” enough to qualify it as part of a takeoff. The takeoff detection method is heavily reliant on observing an increase in average acceleration; however, during activities such as walking or running, the average acceleration is also increased. These activities (walking and running) also significantly increase variance, which makes them easily distinguishable from a user sitting in an aircraft during takeoff (see Figure 5 and Figure 6). Additionally, running increases acceleration to such an extent that it is unlikely to not exceed the upper bounds of both the takeoff roll and liftoff acceleration range, meaning it cannot be detected as takeoff. The “noise filter” was built on the premise that if users are walking or running, it is reasonable to assume that they are not in an aircraft taking off.

Acceleration is considered too “noisy” if the moving variance stays above 3.5 for a minimum of 12 s; thus, the device is considered to not be flying. These values were determined by examining the variance during takeoff and during walking. This threshold value (3.5) was chosen to be in between the variance of these two activities (takeoff and walking), so as to not compromise the performance of the algorithm in cases where the takeoff is very shaky (which would lead to higher variance). The minimum duration is used to filter out sudden short-duration intense shaking that is not caused by walking or running. In the event that acceleration is considered too noisy, the entire 60 s of acceleration data previously mentioned is discarded and not forwarded to the subsequent stage of the algorithm responsible for detecting takeoff. Additionally, the algorithm would not analyze any of the sensor data that were sampled in the following 60 s (i.e., when acceleration is considered too noisy, the next 60 s of sensor data is ignored). This duration was chosen based on the assumption that if a person is walking or running, they are likely not taking off in an aircraft within the next 60 s.

#### 4.1.3. Normalization

After preprocessing and noise filtering, as a final step before detection, the moving average is normalized to compensate for wrongly calibrated accelerometers. On Earth, the standard acceleration due to gravity is $g\approx 9.81\;m/s^{2}$, and a correctly calibrated accelerometer should provide approximately this value when lying on a completely stationary, non-vibrating surface. However, during development, it was observed that some accelerometers reported incorrect values, which in some cases would lead to incorrect or no detection of takeoff.

The normalization step aimed to solve that problem by finding an offset value that is applied to each data point. For example, if the accelerometer reports a magnitude of 9.69 m/s^2^ when stationary, the offset value would be equal to g−9.69=0.12, and the value would be added to each data point of the moving average to compensate for the error. To calculate the offset value, the algorithm first attempts to determine a point in the data where the device is stable. Such a point is determined by identifying when variance is below 0.005 for a minimum of 10 s. If a point meeting these conditions is found, the timestamp of the point is used to find the corresponding acceleration value in the moving average, which in turn is used when calculating the offset value with the formula above. Consequently, changes in gravity due to altitude or local differences on Earth do not influence the detection algorithm.

The values for identifying a stable point (variance below 0.005 for 10 s) were determined by plotting the moving average and moving variance of acceleration from recorded activities (flights, walking, driving, etc., recorded using sensorRecord) in a graph, identifying a point at which the moving average was stable (i.e., the fluctuations in magnitude were minimal, and the moving average was at approximately 9.81 m/s^2^), and observing the value of the moving variance at such a point. The variance must be low for a point to be stable; the moving average theoretically could be stable at an elevated level like in the walking plot in Figure 6 (although one may argue that the moving average is too noisy in this example, it still remains at a stable level overall).

Our normalization algorithm is not perfect and can therefore negatively impact the detection capabilities of the app if the device it is running on has a correctly calibrated accelerometer. Thus, our implementation has an option to disable normalization (see Section 5).

#### 4.1.4. Takeoff Roll Detection

**Figure 7:**
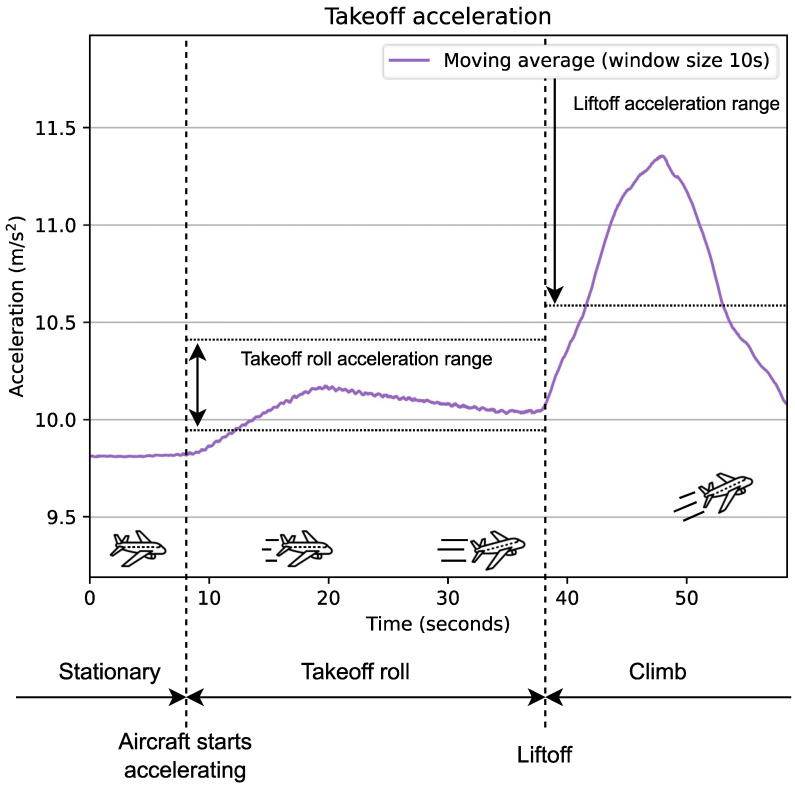
Acceleration during takeoff, shown together with the ranges for detection of takeoff roll and liftoff. The upper bound of the liftoff acceleration range is not visible but is set to 12.0.

Takeoff roll refers to the phase where the aircraft accelerates on the ground, which, according to our observations, lasts roughly 30 s (see Figure 7). For the takeoff roll to be detected, the algorithm requires acceleration to be within the takeoff roll acceleration range, which we defined as being 9.95 ≤ acc ≤ 10.42. If acceleration stays within this range for a minimum of 17 s without exceeding the upper or lower boundaries, the algorithm declares that takeoff roll has been detected. These values were determined by examining the labeled data collected, and observing how long the takeoff roll was (from which we set a minimum time) and how intense the acceleration was (from which we set the takeoff roll acceleration range). A detected takeoff roll is only valid for 41 s and expires if a liftoff is not detected before this time runs out.

Since the retrieved acceleration data have a duration of 60 s, it may often occur that the end of the data is reached while the acceleration is still in the correct range, but before the minimum required time of 17 s has elapsed. Normally, the algorithm always schedules a new analysis 60 s after the previous analysis. However, in this case, the next analysis is instead scheduled 30 s after the previous analysis, half the time of what is typically used. The reason for this is that takeoff detection must be performed with minimal delay, as mentioned in Section 1. If the algorithm believes it might have identified the start of a takeoff roll, there is no reason for it to wait 60 s before having another look. Waiting only 30 s before performing the next analysis could therefore reduce the delay from a takeoff occurs until it is detected by the algorithm.

#### 4.1.5. Liftoff Detection

Liftoff refers to the moment at which the aircraft leaves the ground and becomes airborne and always follows shortly after takeoff roll (unless the takeoff is aborted). The algorithm only attempts to detect liftoff if it has previously detected a takeoff roll (as shown in Figure 4). If liftoff is not detected within 41 s, the detected takeoff roll expires. The algorithm then has to re-detect takeoff roll before it can try detecting liftoff again. This mechanism ensures that if a false positive takeoff roll was produced, a false positive liftoff would not produce a false positive flight unless these events occurred within a short time frame 41 s). The value of 41 s as the maximum allowed delay between takeoff roll and liftoff was determined through experimental observation: examining the labeled data collected and observing the delay between when the acceleration met the conditions required for takeoff roll and when the acceleration met the criteria required for liftoff.

From observing the data collected, it is evident that liftoff was always followed by a sudden spike in acceleration that often reached and sometimes surpassed 11 m/s^2^ for a few seconds (see Figure 7). The exact values could vary greatly and were likely influenced by factors such as aircraft type, takeoff weight, and weather conditions. For liftoff to be detected, the algorithm requires acceleration to be within the liftoff acceleration range, which we defined as being 10.6 ≤ acc ≤ 12.0. If the acceleration stays within this range for a minimum of 5 s without exceeding the upper or lower boundaries, the algorithm declares that liftoff has been detected. As for the takeoff roll detection, these values were determined by examining the labeled data collected and observing the duration of the acceleration spike of liftoff, as well as its intensity. Liftoff detection may also experience that acceleration is within the correct range but the end of the data is reached before the minimum time. In such cases, liftoff detection is carried out as takeoff roll detection is, scheduling the subsequent analysis to occur in 30 s instead of 60.

If both stages of takeoff are detected, the takeoff detection is complete, and the algorithm can determine that the user is flying. When this occurs, as already mentioned, the algorithm stops using the accelerometer and instead starts using the barometer (for landing detection, as described in the next section).

### 4.2. Landing Detection

**Figure 8:**
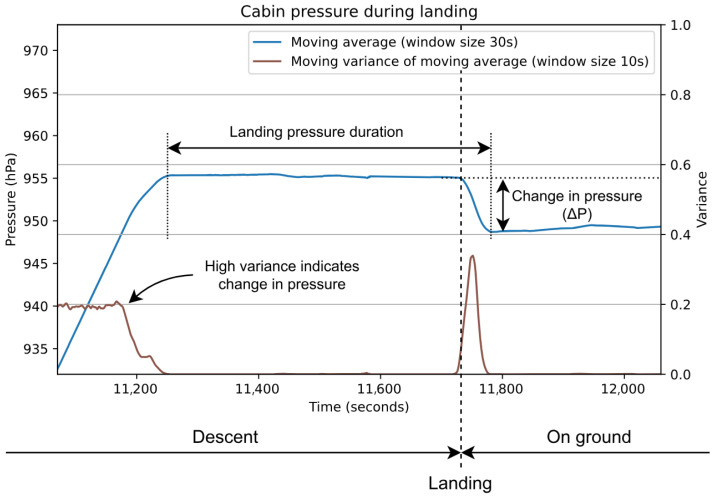
Cabin pressure and variance during landing in an Airbus A321-251N. Here, the pressure is smoothed using moving average, and the moving variance is calculated from the smoothed data.

**Figure 9:**
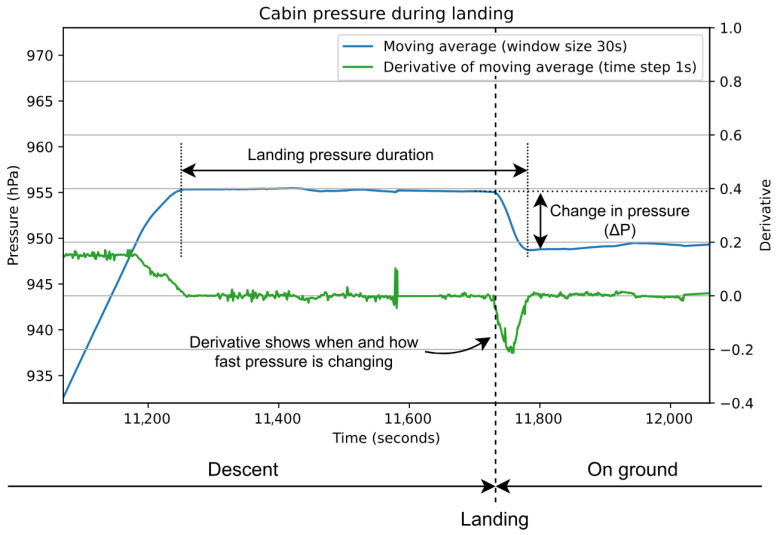
This is the same as in Figure 8, but displaying derivative instead of variance.

For landing detection, the only sensor data used are from the barometer. Using its data, the algorithm calculates the moving average, from which either the moving variance or the derivative is calculated. In fact, there are two options for the landing detection, both calculated from the moving average. Whether the algorithm calculates the moving variance or the derivative is chosen by the user; this is done through the settings page (see Section 5). These approaches are compared in Section 6 and illustrated in Figure 8 and Figure 9.

From examining the collected data, it was clear that there always occurred an increase in pressure when landing that was mostly equalized once the aircraft was on the ground. It is this pressure increase that the landing detection algorithm is based on. The increase in pressure is performed automatically by the cabin pressure controller of the aircraft. Without it, there might occur a sudden unplanned pressure increase caused by the high angle of attack during landing, as well as the ground effect when the aircraft is close to the ground, which may force air in through the outflow valve (which, during normal operations, is used to let air escape from the cabin in a controlled manner) [12,13].

#### 4.2.1. Preprocessing

The preprocessing steps performed in landing detection are quite similar to the ones employed for takeoff detection. The initial step of the algorithm is to retrieve the latest pressure data; however, the quantity of data collected is dependent on the chosen method of landing detection. When using moving variance, the algorithm retrieves 100 s of pressure data, and when using derivative, it retrieves 91 s of pressure data. The data are then used to calculate the moving average with a window size of 30 s. This window truncates the data by its size (as in the preprocessing stage of takeoff detection). The data are later truncated again by either the window size of the moving variance, 10 s, or the time step of the derivative (the time elapsed between the two consecutive points which are used to calculate the derivative), 1 s, depending on the chosen method. Regardless of the method, 60 s of data is analyzed by the algorithm. The initial quantity of data retrieved is different depending on the chosen landing detection method (the goal is to end up with the same duration of data to analyze regardless of the method). All values mentioned here are explained after the next paragraph.

The derivative is calculated for each sample in the data until a timestamp is reached where the time step exceeds the end of the data. This is in practice the same as what occurs in the moving average/variance algorithms; however, the term “window” would be incorrect to use. The reason is that the term “window” suggests there are multiple samples included in the calculation, while the derivative only includes two consecutive samples at a time.

The initial quantity of pressure data retrieved for the algorithm, 100 and 91 s for moving variance and derivative, respectively, was chosen so that a total of 60 s of data remained after all calculations (the duration of the data is truncated by the size of the window/time step), regardless of the chosen landing detection method. A window size of 30 s for the moving average was chosen to achieve a significant smoothing of the pressure data, as this allowed the algorithm to clearly identify where pressure was stable. This was especially important for the derivative method, as it was very sensitive to noise in the data. The moving-variance window size of 10 s was chosen to make the resulting data smoother and less sensitive to small changes, which made the moving-variance detection method more accurate. Similarly, the time step of the derivative is set to 1 s to make it less sensitive to small changes.

The decision to aim for a total of 60 s of pressure data for the analysis, the same duration used for acceleration data, was made because there were no incentives to set this value higher or lower. A duration of 120 s was considered; however, this introduced a delay in landing detection. One could argue that a longer duration would decrease power consumption since it reduces the number of times the algorithm has to retrieve data from the buffer; however, the reduction in power consumption would be negligible.

#### 4.2.2. Detecting Landing Pressure

To detect landing pressure, i.e., the slight increase in pressure before landing, this stage of the algorithm is focused on detecting *pressure plateaus*; these are periods where pressure is stable (by “stable” we mean “not changing”, as in neither increasing nor decreasing). This is accomplished by first iterating through the moving average and checking if the pressure is at least 1.2 hPa above or below the previously detected pressure plateau. This check prevents the algorithm from needlessly attempting to identify a new pressure plateau since the pressure remains at its previously detected stable level. The threshold value of 1.2 hPa was chosen to make room for some slight fluctuations when pressure was otherwise stable and was derived by observing pressure data during flights in the data collected. Additionally, the threshold did not exceed the minimum expected value for ΔP (this value is explained later in this section), as this would break the landing detection algorithm.

If pressure is found to have left its previous stable level, the algorithm attempts to detect a new pressure plateau. How this is accomplished is determined by the chosen method for landing detection (note that this is the only step of the algorithm that is directly influenced by the chosen landing detection method).

**Moving variance**: The algorithm (see Figure 8) calculates the moving variance of the moving average and iterates through it to identify where the variance stays below a threshold value of 0.004 for a minimum of 8.7 s. These values were determined by observing the moving variance where pressure was stable in the collected data.

**Derivative**: The algorithm (see Figure 9) calculates the derivative of the moving average and iterates through it to identify the first absolute value (i.e., the numerical value of a number if the sign is disregarded) that is below a threshold value of 0.015 (no time constraint). This threshold was determined by observing the derivative where pressure was stable.

If the condition of the chosen method is met, the timestamp of this occurrence is used to obtain the corresponding pressure level from the moving average and record a new pressure plateau. This is then compared to the previous pressure plateau to determine if it indicates landing. Two conditions must be met for landing to be indicated: (i) the time elapsed from the start of the previous pressure plateau to the new one (referred to as landing pressure duration, or LPD) must be less than 620 s; and (ii) the pressure difference between the previous and the new pressure plateau (ΔP) must be within the range −23≤ΔP≤−2.

These conditions were determined by observing the duration (LPD) and change in pressure (ΔP) in the landing pressure of flights in the data collected. The ΔP range and maximum LPD were selected to include the great variety these values can have depending on the aircraft type as well as the variability of individual flights. If both conditions are met, the algorithm concludes that the user has landed (thus, no longer flying).

### 4.3. Accelerometer: Other Considerations

The accelerometer [14] is useful for our flyDetect solution since airplanes experience significant acceleration to reach the high speeds required to become airborne.

The alternative to using the accelerometer would be to use location services to determine velocity. This can be achieved by determining the location of the device twice with some delay, calculating the distance between the two locations, and dividing the distance by the time elapsed between the first and second locations. However, this method does have significant drawbacks, (i.e., GPS, WiFi, or cellular).

Using GPS would likely yield the best results for this approach; however, its power consumption is significant, which makes it unsuitable for this solution [11], and, as mentioned earlier, GPS signal reception is somewhat unreliable and inaccurate inside an aircraft. Typically, there is no WiFi coverage on or near runaways. It is true that WiFi is less power-hungry but also less accurate, and it requires external infrastructure which may not be available during takeoff [11]. Finally, cellular-based location detection is even less power-hungry, but its accuracy is further reduced—this approach would require external infrastructure [11]. The accelerometer is one of the least power-hungry sensors, making it a more suitable choice for this use case [1].

### 4.4. Barometer: Other Considerations

The barometer is a slightly less common sensor in smartphones [14] that measures pressure. The air pressure pattern in a pressurized airplane cabin during flight is quite unique, and therefore, the barometer is very suitable for tracking the different phases of a flight. Like the accelerometer, the barometer uses very little power [1]. Additionally, the changes in air pressure occur gradually, which reduces the need for a high sampling rate.

### 4.5. Limitations

The proposed algorithm has some limitations that could potentially cause problems with its ability to successfully detect flights. These are discussed in the subsequent sections addressing (1) the late equalization of pressure, (2) false positives in landing detection, (3) false negatives in landing detection, and (4) restrictions in aircraft types. Several of these limitations are related to incorrect detection or no detection of landing. To help mitigate this problem, our implementation of flyDetect includes a button accessible to the user that forces the algorithm to switch states (i.e., force the algorithm into the “flying” or “not flying” state) as presented in Section 5.

#### 4.5.1. Late Equalization of Pressure

The collected flight data showed significant variations in both the landing pressure duration (LPD) and change in pressure (ΔP) as seen in Table 1. These values fluctuated for each flight but were most notably influenced by the aircraft type. The collected data revealed that most aircraft equalized the cabin pressure with the outside pressure once they landed; however, in a recorded flight with a Boeing 767-300 (B763), the cabin pressure was not equalized until the aircraft had parked. This is inconsistent with the Flight Crew Operations Manual (FCOM) for the Boeing 767-300, which states that the aircraft should depressurize as soon as it touches the ground (touchdown) [15]. Therefore, it is possible that this occurrence was an anomaly; however, this remains unclear as the collected data only contained a single flight with a Boeing 767-300. Nevertheless, this can present a challenge for the algorithm, as taxiing from the runway to the parking spot at a large or busy airport may take a long time, and the algorithm requires the pressure to equalize within a time constraint (620 s) to successfully detect landing. Note that if the time constraint is exceeded, the landing will not be detected.

#### 4.5.2. False Positives in Landing Detection

The cabin pressure and altitude of an aircraft are closely related, with the cabin pressure graph often visually resembling the aircraft altitude graph when flipped vertically (i.e., there is an inverse relationship between aircraft altitude and cabin pressure). In essence, the algorithm for landing is based on detecting two specific pressure changes in relatively quick succession. However, this pattern can also be replicated during a flight due to altitude changes. Although it was not observed in any of the recorded data, an aircraft may decrease altitude for a short period before climbing again during a flight. This may cause the cabin pressure to increase, before decreasing again once the aircraft starts climbing. If the difference in cabin pressure (ΔP) is in the correct range and the altitude changes occur within the specified time constraint of the landing detection algorithm, this would lead to a false positive landing.

#### 4.5.3. False Negatives in Landing Detection

For the algorithm to change state, it is required that either a takeoff or a landing is detected (see Figure 3). As a consequence, it is possible that the algorithm becomes stuck in the “flying” state if takeoff is detected appropriately but landing is not detected. This would be inconvenient to the user; however, the conditions to detect landing may be met outside of an aircraft at a later time. A possible scenario where this could happen is if the user takes an elevator or otherwise descends a few stories of a building and ascends them again shortly after. This would lead to an increase in pressure as the user descends, and going back up would cause the pressure to decrease again.

#### 4.5.4. Restriction in Aircraft Types

The algorithm is restricted in what aircraft types it can detect flight in. It is designed to detect the significant acceleration that occurs during takeoff in common airliners. For example, among the flights recorded, one flight was captured in a De Havilland Canada Dash 8, which is a turboprop aircraft. The recorded acceleration of this flight was significantly lower during takeoff, and therefore not detectable with the selected values for takeoff acceleration (acceleration must be in 9.95 ≤ acc ≤ 10.42 for a minimum of 17 s to qualify as takeoff roll). Additionally, the landing pressure duration (LPD) during this flight was only 7 s, which is significantly shorter than the typical 50 s or more that is usually observed in jetliners. This presents an additional challenge for the algorithm to detect landing, if the moving-variance method is used, as it requires pressure to be stable for a longer period of time to be able to detect the pressure plateau. While it may be possible to update the values used for detection in the algorithm to accommodate for the lower acceleration and shorter LPD (as seen in Section 5), this would increase the likelihood of false positives for both takeoff and landing.

## 5. Implementation

We provide an overview of how sensorRecord and flyDetect were implemented in Android. We begin by providing some information on the minimum SDK and the chosen programming language for the app. Then, we describe the sensorRecord app and its user interface. Subsequently, for flyDetect, we explain how sensor data can be injected for testing and evaluation purposes. We explain the process of defining the different parameters of the flyDetect algorithm and then briefly discuss the user interface of the app. Finally, we discuss device sleep and how this influences both sensorRecord and flyDetect.

### 5.1. Overview

Both sensorRecord and flyDetect were implemented with the minimum SDK set to API level 26, which corresponds to Android 8.0 Oreo. The minimum SDK was chosen as a trade-off between device compatibility and development effort. Choosing a lower API level allows more devices to run the app; however, a lower API level also means having to create workarounds to use features introduced in more recent API levels [16]. API level 26 is a good trade-off where 90.7% of devices are compatible; going lower would need more work for very little gain.

Both apps were written in Kotlin [17], which has been the recommended programming language for Android applications since 2019. The code for both apps is open source and available. The links are the following: for sensorRecord, https://github.com/jonasre/-SensorRecord, (accessed on 15 September 2024), and for flyDetect, https://github.com/jonasre/flyDetect, (accessed on 15 September 2024).

The core functionality of sensorRecord and flyDetect were both implemented as foreground services (according to Android terminology). Both apps display a notification to the user when active, since Android requires foreground services to do so [18]. Tapping the notification opens the respective app. For flyDetect, when the state of the algorithm is switched, i.e., when takeoff or landing is detected, or the state is forcibly changed by the user (more details later), a broadcast is sent out. Other apps can listen to these broadcasts and perform any action accordingly.

### 5.2. How sensorRecord Works

The recording process (with the sensorRecord app) starts when the user presses a button on the user interface. The app then records sensor data from the accelerometer and barometer at a requested sampling frequency of 60 Hz and 4 Hz, respectively. These frequencies were chosen based on assumptions made early about the sampling frequencies required to perform flight detection successfully.

As the sensors generate samples, the timestamp and value(s) of each sample are extracted and converted to a string (text). This string is concatenated with another string that temporarily stores (buffers) the samples, all of them separated with a newline character (“\n”). As soon as the length of the buffer string exceeds 40,000 bytes, the buffer is flushed and written to a temporary file that stores all samples in the recording. This cycle repeats until the recording is stopped by the user. While this cycle runs in the background, the user can add markers (labels) for specific events that occur during the flight. These markers consist of a title and a timestamp, the last of which is determined automatically by the app. Markers are stored in a list until the recording is stopped.

When the recording is stopped, a new file is generated containing the markers at the beginning, followed by the samples stored in the temporary file that is built during the recording. Once the new file is complete, the temporary file can be discarded. The algorithm of sensorRecord can be seen in Figure 2.

#### 5.2.1. Missing Sensors

Not all devices are equipped with a barometer. When such a device uses sensorRecord, the app recognizes that the sensor is missing and proceeds with the recording using only the accelerometer. As a consequence, the resulting recording does not contain pressure data and can therefore not be used for landing detection. However, the recording may still be used to evaluate takeoff, as acceleration data are the only sensor used to detect takeoff. In the exceedingly rare event that neither the accelerometer nor the barometer is available, the recording does not start.

#### 5.2.2. User Interface of sensorRecord

sensorRecord includes a simple user interface divided into two pages: record and history.

The record page lets the user set a title for a recording (i.e., Oslo–London), start and stop the recording, and add markers. Pressing the “stop” button opens a dialog asking users to confirm their intention to stop the recording. Pressing “add marker” on the record page opens a dialog. This dialog lets the user choose an event from a dropdown menu, which is added as a marker in the recording when the user presses “ok”. The events in the dropdown menu are the same as the ones in the instructions, as well as a “custom” event. Selecting “custom” displays a text input field to the user, and when “ok” is pressed, the text in this field is added as a marker in the recording. To make the labeling process easier for the user, the default selected option in the dropdown menu is set as the next expected event (i.e., if the user adds a marker for takeoff, the default selected option will be “landing” next time the “add marker” dialog opens).

The history page displays a list of all saved recordings on the device, each item including the title, size in bytes, and date of the recording. If one of the recordings is pressed, a window opens allowing the user to select a platform to share the file. Recordings can also be deleted by long-pressing on one of them and pressing “delete”.

An additional page containing documentation is available when pressing the question mark in the top right corner of the app; it shows how to record as well as how to share the file once the recording is complete.

### 5.3. Defining the Parameters of the flyDetect Algorithm

The algorithm of flyDetect uses certain parameters when detecting flight. These were determined using experimental observation of sensor data of flights (and other activities) that were collected using sensorRecord. For each parameter, we plotted the relevant data (e.g., acceleration, pressure, moving average/variance, or derivative) in a graph and inspected the duration (x) of the event that the algorithm was trying to detect (e.g., takeoff roll, liftoff, etc.), as well as the y-value of the plotted data during the event. From this, thresholds and ranges were created as part of conditions that must be met for an event to be detected (e.g., acceleration must be in 9.95 ≤ acc ≤ 10.42 for a minimum of 17 s to qualify as takeoff roll).

### 5.4. Sensor Data Injection in flyDetect

A system was included to enable the loading of text files containing sensor data for testing and evaluation purposes. This allows the flyDetect app to disable the physical sensors and instead replay a recording of an activity such as a flight. With this system, the algorithm cannot distinguish between injecting recorded sensor data and receiving measurements from physical sensors in real time.

### 5.5. User Interface of flyDetect

Since flyDetect is supposed to perform most of its work in the background, the user interface provided is limited in complexity, although according to the definition in the Android documentation [19], flyDetect is always in the foreground since it runs a foreground service; however, by “background”, we mean that the app is not visible on the screen, apart from the notification. Still, it provides the user with some means to interact with the underlying algorithm. The user interface consists of 4 pages: home, developer, flight history, and settings.

**Figure 10:**
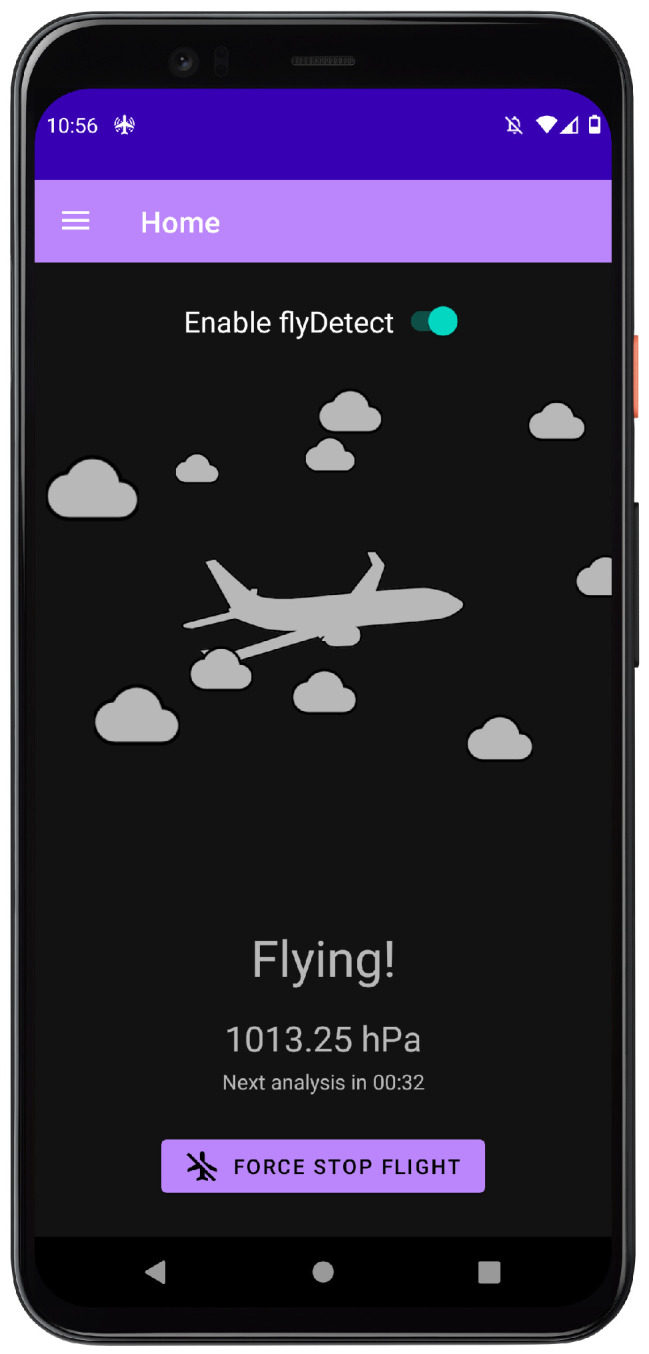
The home page.

The home page (see Figure 10) is where the user can enable/disable flyDetect, view the current state of the algorithm, view the current sensor reading and time until the next analysis, and force switch the state of the algorithm. In particular, this page contains the master switch for enabling or disabling the algorithm. If enabled, this page also displays the current state of the algorithm (flying or not flying), the latest reading from the enabled sensor, and the time in minutes and seconds until the next scheduled analysis of the sensor data (meaning, the time until the algorithm retrieves data from the circular buffer and then attempts to detect takeoff or landing). Additionally, it provides a button that can forcibly switch the state of the algorithm. This functionality is convenient in the event that the algorithm somehow gets stuck in the wrong state (as previously mentioned).

**Figure 11:**
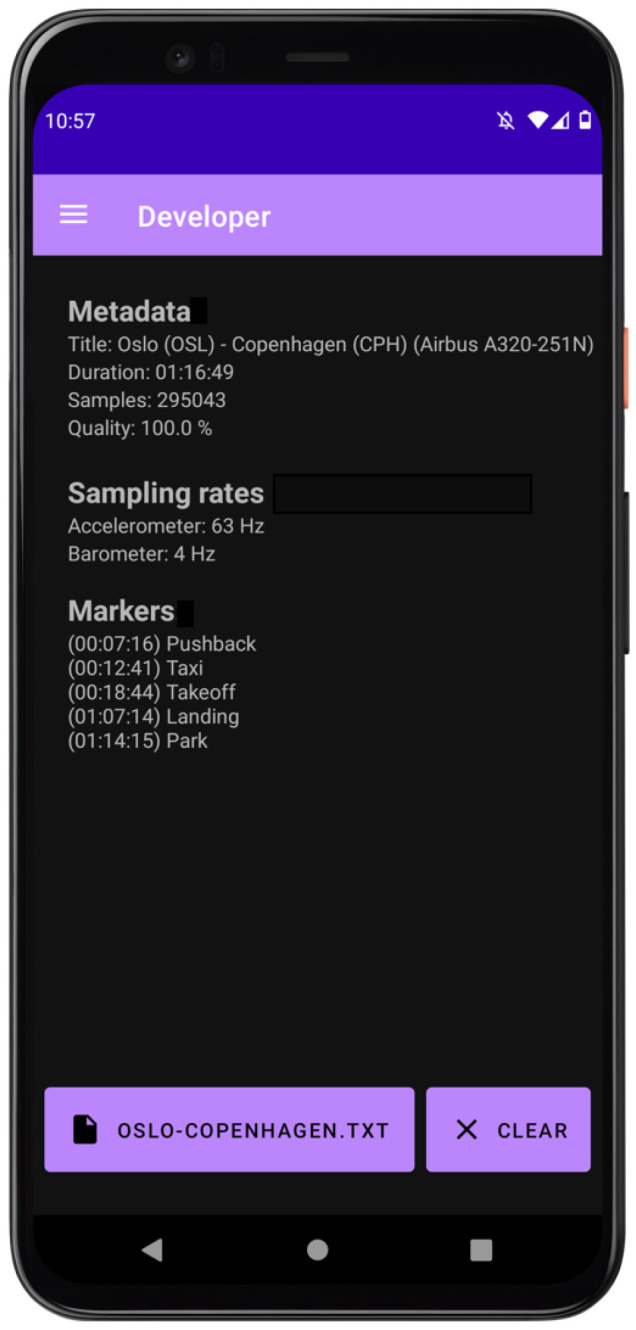
The developer page.

The developer page (see Figure 11) works as an interface for the sensor injection functionality of flyDetect. This page makes it possible to load a file containing sensor data, which the app reads and displays some general information.

**Figure 12:**
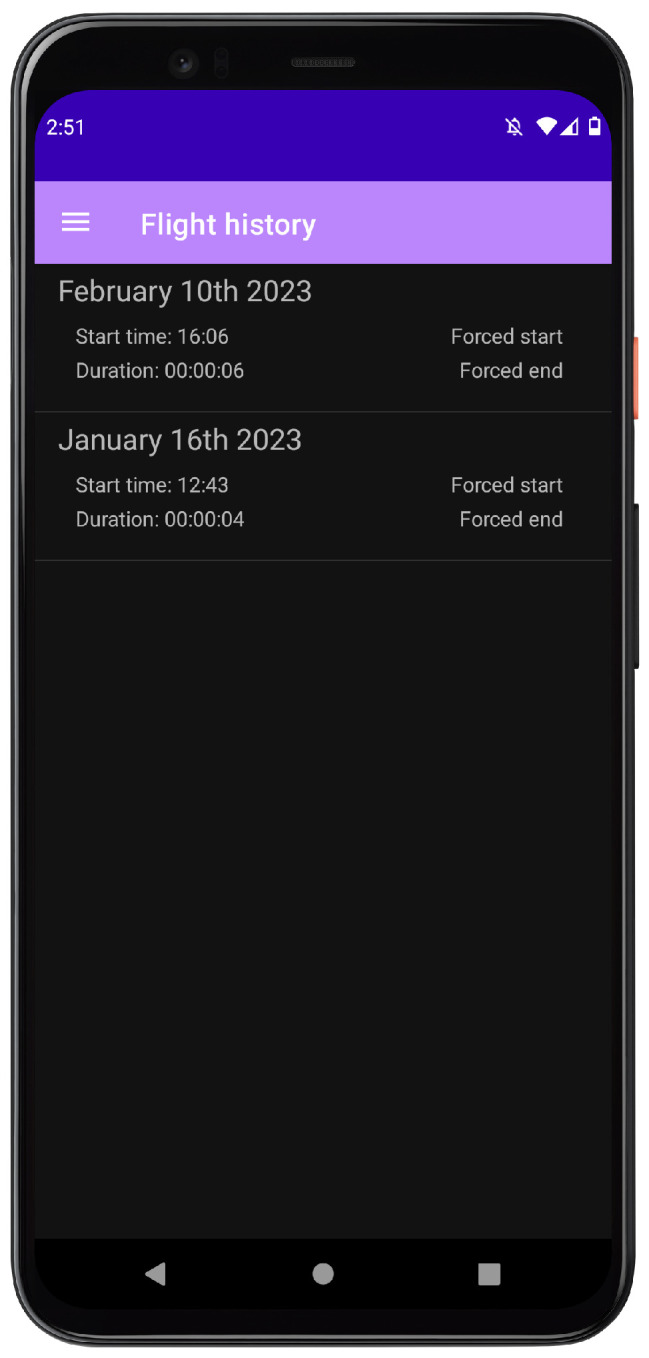
The flight history page.

The flight history page (see Figure 12) displays the date and duration of all flights detected by flyDetect. Additionally, it displays if the flight was forcibly started or ended. The purpose of this page is to make the evaluation easier since it becomes unnecessary to continuously monitor the display to confirm whether the app successfully detected the flight or not (no need to continuously look at the state of the algorithm on the home page). Each flight is stored with the time and date it occurred, as well as the duration of the flight (time elapsed from takeoff to landing). Additionally, this page displays if the flight was forcibly started or ended.

**Figure 13:**
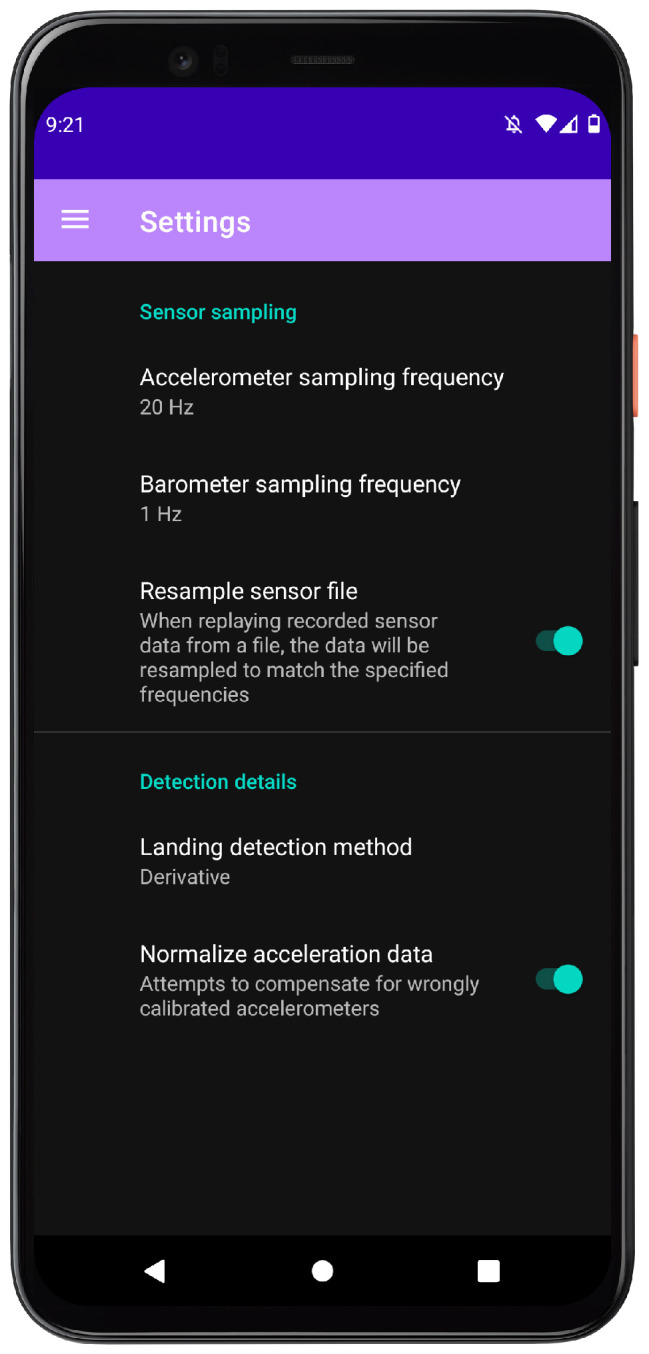
The settings page.

The settings page (see Figure 13) makes it possible to change certain settings (of the flyDetect app): sampling frequencies, enabling or disabling resampling, and landing detection method. These settings are not intended for the user but were included to simplify testing and evaluation: they include changing the sampling frequency of both the accelerometer and the barometer, deciding if sensor data read from a loaded file should be resampled to the specified sampling frequencies or not, and choosing the landing detection method of the algorithm (either moving variance or derivative). A final setting, “Normalize acceleration data”, is included to enable or disable the normalization. This was included because of the potential negative impact this can have on detection if the accelerometer is already correctly calibrated (we discuss this normalization issue in Section 4).

### 5.6. Dealing with Device Sleep and Device Configuration

Android devices enter a sleeping state when the screen turns off, which causes problems for both sensorRecord and flyDetect. In the subsequent sections, we discuss this behavior and how it differs from device to device, as well as how we mitigate the problems it causes.

#### 5.6.1. Device/CPU Sleep

When the screen of an Android device is turned off, the system quickly enters a sleep state [20]. This feature is poorly documented, but from our experiments, we observed that the device did not enter the sleep state if it was plugged in. In the sleeping state, the CPU does not perform any work, which creates issues for both flyDetect and sensorRecord. The issue with this is not only that the CPU would not be analyzing data for flyDetect or not be saving data for sensorRecord, but also that it would be unable to receive samples generated by the physical sensors of the device. An Android device will periodically wake the CPU when the screen is off. In our experiment, each wake usually lasted a bit more than 300 ms, but it occasionally lasted up to 3 s. During each wake, the CPU appeared to continue performing work for apps as normal.

#### 5.6.2. Sensor Batching

Since devices enter a sleep state when the screen is turned off, their CPU will often not be awake to receive samples from the physical sensors as they are generated. Android devices mitigate this problem by using sensor batching [21,22]. This means that the generated sensor data are stored in a hardware buffer (commonly referred to as “FIFO” in the Android documentation [21]) before it is sent to the application. As a result, the physical sensors can continue generating samples while the CPU is asleep and send them to the application later when the CPU wakes up. This is because samples are continuously buffered in the FIFO while the CPU is asleep, which all have to be processed for the CPU to “catch up” with the latest data. To use sensor batching, it is required that the device or sensor possesses a FIFO (these can either be shared by multiple sensors on the device or be dedicated to a single sensor, with each sensor having its own). Without a FIFO, all samples generated while the CPU is asleep are lost. The FIFO works as a circular buffer, which means that if the CPU does not wake up to receive the samples before the FIFO is full, the newest samples overwrite the oldest ones. Note that when only a single sensor is active and the FIFO is shared among multiple sensors, it is common to let the active sensor use the entire FIFO. In these cases, the FIFO is able to buffer a longer duration of sensor data at a time, which in turn means that the device can be sleeping for a longer period of time before data loss occurs.

#### 5.6.3. Differences in Device Configuration

A wake lock is a system feature that lets applications control the power state of the host device [20], while a partial wake lock is a type of wake lock that keeps the CPU on until the partial wake lock is released [23].

**Figure 14:**
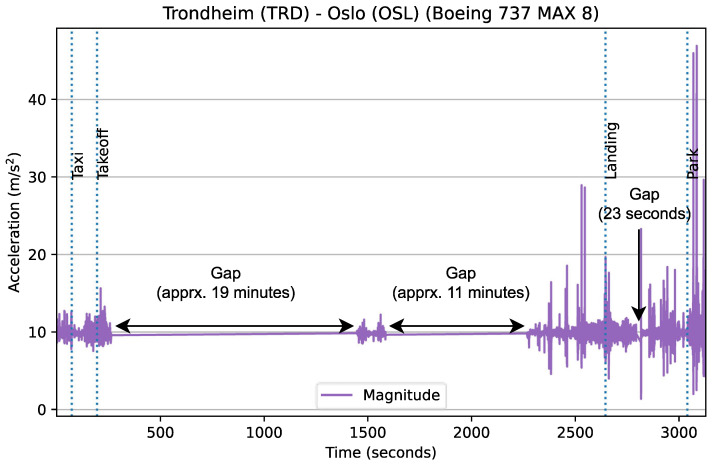
An example of a fragmented flight recording captured with a Huawei P20 Lite. Note the large gaps in the acceleration data. This recording has a quality score of 39.7%.

sensorRecord, the application used for data collection, acquires a partial wake lock, which is held as long as a recording is in progress. However, many of the recorded flights in our dataset still contained small gaps (i.e., if spread across a timeline, there were some time periods that contained no data), and although these gaps typically only had a duration of a few seconds to a minute, this was not always the case. In more extreme instances, some of the recorded flights were fragmented to such an extent that, in reality, the recorded data covered less than 50% of the flight, with the duration of some gaps exceeding 15 min (e.g., see Figure 14). The few severely fragmented recordings in our dataset were generated by the same few devices; therefore, it is evident that there are significant variations in how different devices handle device sleep and wake locks. These variations are likely a result of device configuration.

The developer page of flyDetect displays a calculated quality score for a file containing sensor data. This score describes how much of the recording contains data. If a recording has a score of 100%, then it contains no gaps; however, as the score decreases, the recording gradually becomes more fragmented (at 0%, there are no data whatsoever). For calculating the quality score, we defined a gap as a duration greater than 1 s between two samples; the accelerometer and barometer were sampled at 60 Hz and 4 Hz, respectively, which meant that the duration between two samples should in theory never be greater than 250 ms. The cumulative duration of all gaps was subtracted from the duration of the recording, and then all this was divided by the duration of the recording.

We can compare different devices based on the quality of the recorded data they produce (see Table 2). These recordings were all made using a partial wake lock, as previously mentioned. Bearing this in mind, devices from Huawei stood out in this dataset, sharing an average quality below 50%, lower than devices from any other manufacturer. All Samsung devices had an average quality above 90%, with little variation between the different models. Finally, the Google Pixel 4a and Motorola devices seemed to honor the partial wake lock every time (the Sony Xperia 10 III might have been included in this list, but due to being limited to only one recording, its behavior could not be confirmed with certainty). It is reasonable to assume that some device manufacturers introduce their own aggressive power optimizations to extend battery life, which causes this kind of behavior.

#### 5.6.4. Our Approach

sensorRecord includes a partial wake lock to solve the problem of device sleep. We could have included a partial wake lock in flyDetect (like in sensorRecord) but this was avoided for the following reason:

- Partial wake locks can significantly impact the battery life of the device [20]. The requirements for our solution (defined in Section 1) emphasized the importance of low power consumption, which made the addition of a (partial) wake lock unsuitable.
- Partial wake locks seem to be unnecessary. The data collection app was tested without a partial wake lock on a Google Pixel 4a 5G at nighttime. The test was performed five times and was approximately 8 h long each time. When running, the device was stationary, unplugged, and without user interaction, and still achieved a median quality score of 96.6%. Although a partial wake lock would increase this quality score to 100% (this was tested and verified with the same device), it would certainly come at a much higher cost in terms of power consumption.
- Many devices do not honor partial wake locks. This means that devices that do honor partial wake locks would see a slight increase in quality and a large increase in power consumption, while devices that mostly ignore partial wake locks (like the Huawei devices in our dataset) would likely achieve the same quality as in Table 2, which is already too low to detect flight reliably.

## 6. Evaluation

We now show the results while evaluating our solution.

### 6.1. Data Collection

The data collection process was distributed among volunteers. In total, 34 flight recordings were collected from 11 volunteers. Of these recordings, 13 different aircraft were included (although several aircraft in the dataset were closely related, for instance, the Airbus A320-232 and Airbus A320-251N). One was excluded from the evaluation because the aircraft was a *De Havilland Canada Dash 8*, which is a turboprop aircraft (turboprops cannot be detected reliably as mentioned in Section 4), and two were excluded because they contained gaps covering the takeoff (acceleration data for takeoff were missing). Consequently, 31 recordings were used for the evaluation.

Unfortunately, not all devices that contributed to the data collection had barometers, which led to only 23 recordings including pressure data. To detect landing, the algorithm requires pressure data (as explained in Section 4), which means that the entirety of the algorithm (takeoff and landing detection) could only be tested with the 23 recordings that contained such data. However, the remaining recordings could still be used to evaluate takeoff detection, since all of them contained acceleration data which the algorithm requires.

As visible in Table 3, the majority of the collected data were recorded by young volunteers (the age among the volunteers ranged from 20 to 64). It is unclear how the data collected were influenced by the age of the data collectors; however, the impact was likely negligible. The reason for this is that our data collection app, sensorRecord, requires minimal interaction with the user, which minimizes the impact of the technological familiarity of the user. Poor technological familiarity may lead to markers being set too early or too late; nevertheless, from observing our dataset, markers seemed to be set at the correct times for takeoff and landing. These two were easily recognizable by visually inspecting a graph of acceleration and pressure.

### 6.2. Approach

The following aspects were evaluated:

1. **Accuracy of flyDetect:** a percentage of how many of the recorded flights the app was successfully able to detect;
2. **Detection time of flyDetect**: the time elapsed from when takeoff/landing occurs until it is detected;
3. **Power consumption of flyDetect:** power consumed in milliamperes, and how many percent of the battery is drained per hour.

The evaluation of the accuracy and detection time of flyDetect was carried out in an emulator and took advantage of the sensor injection feature of the app (mentioned in Section 6). This allowed previously recorded data to be injected into the algorithm, instead of requiring a physical device to go on a flight to evaluate the app. Not only flight data were tested, but other activities such as driving, walking, train rides, etc., were also included to check for potential false positives. The activities included in the test data had a cumulative duration of almost 18.5 h and are listed in Table 4. Evaluating accuracy and detection time followed the same steps: (1) load a recording of a flight or another activity and enable flyDetect and (2) observe if flyDetect detected anything; if there was a true positive, then observe the detection time.

### 6.3. Accuracy of flyDetect

In the subsequent sections, we evaluated the accuracy of flyDetect in two parts: takeoff and landing.

#### 6.3.1. Takeoff Detection

We evaluated the accuracy of takeoff detection by running the 31 recorded flights (mentioned previously) through flyDetect. This was achieved with different settings enabled: we first experimented with different sampling frequencies for the accelerometer (10 Hz, 15 Hz, 20 Hz, and 30 Hz), which is the only sensor used for takeoff detection. Also, we exercised enabling and disabling the normalization stage of the algorithm.

The results (displayed in Table 5) showed that increasing the sampling frequency of the accelerometer beyond 20 Hz had no effect on the accuracy of takeoff detection. It also showed that the accuracy differed depending on whether normalization was enabled or disabled. In our dataset, two of the devices used for recording had incorrectly calibrated accelerometers, which caused three of their combined recorded flights to be dependent on normalization to successfully detect takeoff. These were the only recordings in which takeoff was not detected when normalization was disabled (resulting in an accuracy of 90.3%). When normalization was enabled, takeoff was successfully detected in these recordings; however, the normalization made takeoff undetectable in two other recordings (resulting in 93.5% accuracy). This is why normalization can be toggled in the settings of flyDetect as it can negatively impact the detection performance if the accelerometer is correctly calibrated. When we excluded the recordings where the accelerometer was incorrectly calibrated, the accuracy of flyDetect was 100%.

The last row in Table 5 shows the detection accuracy with “Dynamic” normalization. This showed that flyDetect could detect takeoff in all recorded flights when the accelerometer sampling frequency was 15 Hz or greater. However, we would recommend running flyDetect with an accelerometer sampling frequency of 20 Hz, as it sometimes occurred that some flights were dependent on normalization to detect takeoff at both 10 Hz and 15 Hz. We do not recommend increasing the sampling frequency above 20 Hz since this did not seem to improve accuracy, and higher frequencies caused higher power consumption.

We also tested flyDetect with almost 18.5 h of other activities including traveling by car, train, and walking (for the full list, see Table 4). During these tests, we ran flyDetect with the same combination of settings as when testing with flight recordings (i.e., accelerometer sampled at 10 Hz, 15 Hz, 20 Hz, and 30 Hz, and normalization both enabled and disabled). The results showed that flyDetect produced no false positives regardless of configuration in any of the test data (no table is included since no false positives were produced). It did occur that flyDetect detected takeoff rolls, especially in recordings of traveling by car. However, this was only one stage of the takeoff detection algorithm, and since it did not detect a liftoff shortly after, the takeoff roll was considered a false positive and discarded. flyDetect did therefore not count this as a takeoff, thus having an accuracy of 100% for takeoff detection.

#### 6.3.2. Landing Detection

When evaluating the accuracy of landing detection, we ran the 23 recorded flights that contained pressure data through flyDetect. Each flight was tested with all possible combinations of these settings: barometer sampling frequency at 1 Hz, 2 Hz, or 4 Hz, and landing detection method set to derivative or moving variance. We did not test frequencies below 1 Hz because this was the lowest possible sampling frequency for the barometer on a Google Pixel 4a 5G (which is the device we used). We expected other barometers to have similar minimum sampling frequencies.

**Figure 15:**
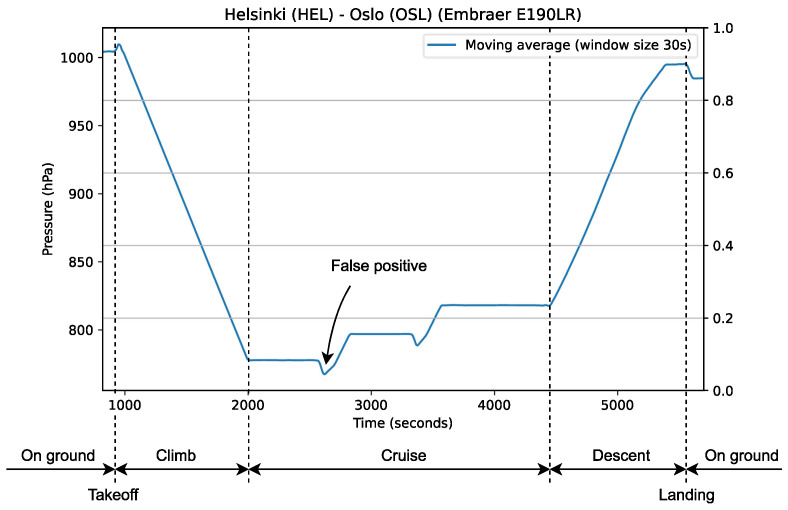
Pressure during the recording which causes a false positive when using the derivative landing detection method.

The results (displayed in Table 6) showed little variation in accuracy across the different sampling frequencies. It also showed that the derivative landing detection method achieved the highest accuracy with 95.7% (22 of 23 flights) on all tested sampling frequencies. The recording that reduced the accuracy from 100% to 95.7% possessed a unique pattern in its pressure: when the altitude was reduced during the cruising phase, pressure was briefly decreased before increasing again (see Figure 15). The derivative method detected the lowest point in this decrease as a pressure plateau, and since the duration between this and the previous plateau (LPD) was shorter than the maximum duration and the change in pressure (ΔP) was within the correct range, the algorithm detected this as a landing (pressure plateaus and the values mentioned here are explained in Section 4). It was unclear if these pressure changes were typical for the aircraft in this recording (Embraer E190LR) as our dataset only contained two recordings from it (the other recording did not show this behavior; however, neither did it change altitude during the cruising phase). This behavior was not observed in any other recordings in our dataset.

When the moving-variance method was unable to detect landing, it was usually because it was unable to detect the pressure plateau when pressure increases before landing (again, more details about landing detection in Section 4). However, it was capable of correctly detecting landing in the recording discussed in the previous paragraph (and which is displayed in Figure 11. This is because the derivative method is more sensitive to pressure plateaus than the moving-variance method. In the aforementioned recording, the pressure was not stable when it dipped (marked on the figure with “False positive”) and thus not detected as a plateau.

We assessed that to run flyDetect with optimal landing detection accuracy, the derivative method should be used along with a barometer sampling frequency of 1 Hz. We do not recommend sampling frequencies above 1 Hz, as this did not improve accuracy (for the derivative method), and a higher frequency causes a higher power consumption.

### 6.4. Detection Time of flyDetect

While evaluating the accuracy of flyDetect, we also recorded the detection time, i.e., the time from when takeoff/landing occurred until it was detected. To find out when these events occurred, we relied on the markers saved in the recording generated by sensorRecord. The instruction of when to mark takeoff was “when the aircraft is on the runway and you hear the engines increase power, i.e., not when the aircraft lifts off, but when it starts accelerating”. For landing, the instruction was to mark it “the second the aircraft touches the ground again”. As previously mentioned, the markers for these events in our dataset seemed to be set correctly, since takeoff and landing were easily identifiable by observing a chart of each recording where acceleration and pressure were plotted. Using these markers, we included three duration measurements: (i) the duration from the marker until the event was detectable, i.e., the time it took from the timestamp of the marker until the timestamp of the final sample flyDetect uses to detect the event; (ii) the duration from when the event was detectable until the event was detected, i.e., from the timestamp of the final sample flyDetect uses to detect the event until the timestamp of the last sample included in the retrieved window of data; and (iii) the duration from the marker until the event was detected, i.e., the cumulative duration of the two periods previously mentioned.

#### 6.4.1. Detection Time of Takeoff Detection

For takeoff detection, across all tested settings, the duration from when the takeoff started until it was detectable (i) was 35 s on average. The duration from when takeoff was detectable until it was detected was (ii) 26 s on average. The duration from when the takeoff started until it was detected was (iii) 61 s on average (or 1 min and 1 s). This detection time is fine (e.g., for enabling airplane mode on the device as a last resort). We discovered no trends in any configuration of flyDetect leading to a lower or higher detection time. In fact, the detection time was mostly unchanged when running the same recording with different settings.

#### 6.4.2. Detection Time of Landing Detection

For landing detection, we divided the results into two parts: one for the moving-variance landing detection method and one for the derivative landing detection method. For the moving-variance method, the duration from when landing occurred until it was detectable (i) was 57 s on average. The duration from when landing was detectable until it was detected (ii) was 63 s on average (or 1 min and 3 s). The duration from when landing occurred until it was detected (iii) was 120 s on average (or exactly 2 min).

For the derivative method, the duration from when landing occurred until it was detectable (i) was 37 s on average. The duration from when landing was detectable until it was detected (ii) was 56 s on average. The duration from when landing occurred until it was detected (iii) was 93 s on average (or 1 min and 33 s).

The results showed that the derivative landing detection method was better than the moving-variance method in detection time, with the moving variance requiring on average 27.7% more time to detect landing.

When evaluating the detection time of landing detection, we excluded one recording. This recording contained a flight where the cabin pressure was not equalized until the aircraft had parked (in all other flights in our dataset, cabin pressure was equalized once the aircraft landed). The behavior observed in the aforementioned flight was not consistent with the Flight Crew Operations Manual of the aircraft [15]. Because of this, the landing was not detectable until 7 min and 47 s after it occurred (with the derivative method; the moving-variance method could detect landing after 7 min and 52 s). We excluded this recording because the results it generated were not representative of the actual performance of the app.

### 6.5. Power Consumption

The evaluation of power consumption was carried out with a physical device. For flyDetect, this involved using different sampling frequencies for the sensors, as well as running it with the algorithm in different states, i.e., both “flying” and “not flying”. To evaluate power consumption, Battery Historian, a piece of software created by Google [24], was used to inspect how much power was drained on a device. Battery Historian takes a bug report generated by a device and analyzes it to provide an overview of what apps were running at what time, when the CPU was running, the battery level, when the device was in Doze mode (a state where certain battery optimizations are introduced [25]), etc.

The device utilized for these tests was a two-year-old Google Pixel 4a 5G with a battery capacity of 3885 mAh. However, from observing data from the device in Battery Historian, the battery capacity seemed to have degraded to approximately 3550 mAh. Otherwise, the battery appeared healthy, with a full charge often lasting two days with normal usage. The actual longevity of a full charge is determined by how much the device is used and the power consumption of the activities performed. Additionally, “normal usage” is subjective; a sufficiently active user would certainly be capable of draining the battery in a day.

We also measured the power consumption of the device by measuring the remaining charge in the battery after starting the target app and then measuring the remaining charge again later when the app had been running for a while. We also determined the power consumption in percentage per hour and the power consumption (further converted to hours of battery life) using the maximum capacity of the battery (3550 mAh).

When collecting power consumption measurements, we encountered high variability in some data. This high variability was likely caused by other apps running. Because of this, we used the median instead of the mean to get more representative measurements. Although the mean could be used for many of the values, we used the median for all measurements for consistency.

#### 6.5.1. Power Consumption of flyDetect

To evaluate the power consumption of flyDetect, the following tests were performed: (i) algorithm in the “not flying” state, accelerometer sampled at 20 Hz, and normalization enabled; (ii) algorithm in the “flying” state, barometer sampled at 1 Hz, moving-variance landing detection method; and (iii) algorithm in the “flying” state, barometer sampled at 1 Hz, derivative landing detection method. The tests were performed at night to minimize unexpected additional power consumption as it reduced the probability of incoming calls or messages since most people (who are somewhat likely to call or send messages) are asleep, and the user is not using the device. Each test lasted approximately 8 h and was repeated five times. The reason for repeating the tests was to make the results more stable (more representative of the actual power consumption). We present the results in the following sections.

#### 6.5.2. Not Flying, 20 Hz Accelerometer, Normalization Enabled

**Figure 16:**
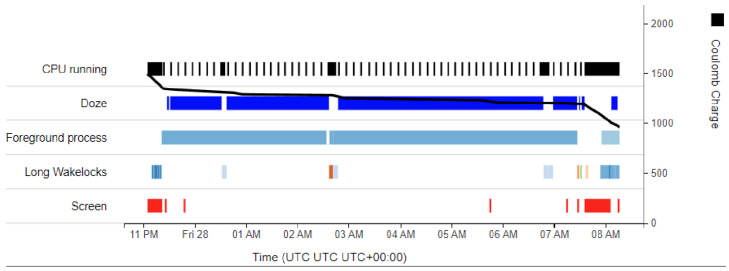
Power consumption of flyDetect in “not flying” state (screenshot from Battery Historian). The black line at the top shows the charge remaining on the battery in milliampere-hours (mAh). The bars in the chart indicate when something is enabled/active/on. “CPU running” shows when the CPU is active. “Doze” shows when Doze mode is enabled. “Foreground process” displays when the target app is active. “Long Wakelocks” show wake locks (including partial wake locks) that exceed one minute in duration (the colors on this indicate which apps hold wake locks (which is often more than one); however, this information is not relevant for our purposes). “Screen” shows when the screen is on.

This section evaluates the power consumption of flyDetect when the algorithm was in the “not flying” state, sampling the accelerometer at 20 Hz and with normalization enabled (see a screenshot from Battery Historian in Figure 16). From the results of the accuracy, evaluated previously, we recommend this configuration when using flyDetect, although normalization should be disabled if the accelerometer of the device is correctly calibrated. We expected that if normalization was disabled, power consumption would likely be slightly lower. This was because normalization involved iterating through the sensor data twice, once to find an offset value and once to apply the offset value to the data (details in Section 4). The test results (listed in Table 7) revealed that when running flyDetect in this configuration, the entire device had a median power consumption of 18.22 mA, which is equal to 0.51%/h or approximately 8 days of battery life. Battery Historian estimated that flyDetect was responsible for only 0.40 mA (0.01%/h).This power consumption is low enough that a user would not mind having flyDetect running continuously on the device.

#### 6.5.3. Flying, 1 Hz Barometer, Moving Variance

This section evaluates the power consumption of flyDetect when the algorithm was in the “flying” state, sampling the barometer at 1 Hz and using the moving-variance landing detection method (no Battery Historian screenshot is included since it would look almost identical to Figure 16). This configuration of flyDetect had a lower accuracy and higher detection time compared to the derivative landing detection method.

The test results (listed in Table 8) revealed that when running flyDetect in this configuration, the entire device had a median power consumption of 19.67 mA, which is equal to 0.55%/h or approximately 7.5 days of battery life. Battery Historian estimated that flyDetect was responsible for 0.87 mA (0.02%/h) approximately, twice the power consumption when in the “not flying” state. This increase was caused by differences in power consumption between the accelerometer and barometer on the test device. By using the getPower() method on these sensors in the code, we observed that the accelerometer had a power consumption of 0.17 mA, while the barometer had a power consumption of 0.70 mA [26].

#### 6.5.4. Flying, 1 Hz Barometer, Derivative

This section evaluates the power consumption of flyDetect when the algorithm was in the “flying” state, sampling the barometer at 1 Hz and using the derivative landing detection method (no Battery Historian screenshot is included since, as in the previous section, it would look almost identical to Figure 16). The test results (listed in Table 9) revealed that when running flyDetect in this configuration, the entire device had a median power consumption of 16.55 mA, which is equal to 0.47%/h or almost 9 days of battery life. Battery Historian estimated that flyDetect was responsible for 0.84 mA (0.02%/h). The power consumption when using the derivative landing detection method was slightly lower compared to the moving-variance method. This was caused by the moving variance being slightly more computationally expensive to calculate than the derivative.

#### 6.5.5. Conclusions

The results from these tests of flyDetect, when the algorithm was in the “not flying” state (see Table 7) and the app was running in the recommended configuration, showed that a power consumption of 0.40 mA (0.01%/h) was achieved. Considering the app would mostly be in the “not flying” state (since the majority of users likely spend more time on the ground than they do in the air), this was a satisfactory result; it was an acceptable power consumption for a user even though flyDetect would be running continuously.

In the “flying” state, sampling the barometer at 1 Hz, the moving-variance landing detection method achieved a power consumption of 0.87 mA (0.02%/h) compared to the 0.84 mA (0.02%/h) achieved with the derivative method (see Table 8 and Table 9, respectively). We assessed that the moving-variance method used slightly more power because the moving variance is computationally more expensive to calculate than the derivative. The increased power consumption in both methods compared to when flyDetect is in the “not flying” state was caused by the barometer having a higher power consumption than the accelerometer on the test device.

## 7. Conclusions

The goal of this work was to develop a method for detecting flight in near real time and validating the method by developing a flight detection app for Android. The motivation was that such a detection system could potentially be used to automatically manage airplane mode on smartphones, reducing the risk of interference with aircraft systems if users forget or are otherwise unable to enable airplane mode themselves.

This paper proposed flyDetect, an Android system capable of detecting the beginning and end of a flight in near real time. The detection was performed locally on the device, independent of external sources of information and used only the accelerometer and barometer sensors. To detect takeoff, the algorithm looked for the substantial increase in acceleration which occurs when an aircraft takes off. To detect landing, the algorithm looked for the increase in pressure which occurs when landing.

## Figures and Tables

**Table 1:** Statistics showing average landing pressure duration (LPD) and change in pressure ΔP. Grouped by aircraft type.

Aircraft Type (ICAO Code)	Number of Flight Recordings	${\mathrm{LPD}}_{\mathrm{avg}}$	$\Delta P_{\mathrm{avg}}$
**A20N**	4	200	6.5
**A21N**	1	520	6
**A320**	2	215	8
**A321**	1	210	5.5
**B38M**	2	40	15
**B738**	9	60.56	9.78
**B753**	1	280	2.5
**B763**	1	600	3
**E190**	2	140	9.5
**E290**	1	40	10
**Total/Average**	**24**	**157.71**	**8.58**

**Table 2:** This table displays, for each device, the average quality of all of its recordings, the number of recorded flights from this device in the dataset, and if the device has a barometer or not.

Device	Average Quality	Number of Flights	Barometer
Google Pixel 4a	100	4	Yes
Huawei P20 Lite	43.8	3	No
Huawei P30 Pro	28.9	1	No
Motorola Edge 30	100	2	No
Motorola Moto G100	100	2	No
Samsung Galaxy S10+	98.08	6	Yes
Samsung Galaxy S21	98.98	4	Yes
Samsung Galaxy S21 Ultra 5G	97.36	5	Yes
Samsung Galaxy S22	97.1	4	Yes
Samsung Galaxy S7 Edge	94.3	2	Yes
Sony Xperia 10 III	100	1	No

**Table 3:** Age range by mode of transportation.

Age Range	Number of Volunteers	Number of Flights
20–24	4	13
25–29	3	7
30–35	2	4
55–59	1	6
60–64	1	4
**Total**	11	34

**Table 4:** Activities carried out by mode of transportation.

Activity	Duration
Bus	00:50:01
Car	04:06:07
Idle	07:29:51
Metro	00:16:42
Skiing (cross country)	02:16:26
Train	01:23:51
Walking	02:01:20
**Total**	18:24:18

**Table 5:** Takeoff detection accuracy.

Normalization	Sampling Frequency			
	10 Hz	15 Hz	20 Hz	30 Hz
**Disabled**	74.2	87.1	90.3	90.3
**Enabled**	87.1	93.5	93.5	93.5
**Dynamic**	93.5	100.0	100.0	100.0

**Table 6:** Landing detection accuracy.

Detection Method	Sampling Frequency		
	1 Hz	2 Hz	4 Hz
**Moving variance**	87.0	91.3	91.3
**Derivative**	95.7	95.7	95.7

**Table 7:** Results from running flyDetect in the “not flying” state with the accelerometer sampled at 20 Hz and normalization enabled.

		Power Device		Power App (BatHist est.)	
ID	Duration	mA	%/h	mA	%/h
1	08:01:29	18.22	0.51	0.44	0.01
2	08:04:24	17.20	0.48	0.40	0.01
3	08:52:11	18.49	0.52	0.32	0.01
4	07:58:39	17.29	0.49	0.49	0.01
5	08:05:24	18.53	0.52	0.31	0.01
**Med.**	**08:04:24**	**18.22**	**0.51**	**0.40**	**0.01**

**Table 8:** Results from running flyDetect in the “flying” state with the barometer sampled at 1 Hz and using the moving-variance landing detection method.

		Power Device		Power App (BatHist est.)	
ID	Duration	mA	%/h	mA	%/h
1	07:45:36	15.49	0.44	0.87	0.02
2	07:40:59	22.46	0.63	0.88	0.02
3	08:30:19	27.64	0.78	0.88	0.02
4	08:08:08	19.67	0.55	0.87	0.02
5	09:07:02	16.09	0.45	0.82	0.02
**Med.**	**08:08:08**	**19.67**	**0.55**	**0.87**	**0.02**

**Table 9:** Power results from running flyDetect in the “flying” state with the barometer sampled at 1 Hz and using the derivative landing detection method.

		Power Device		Power App (BatHist est.)	
ID	Duration	mA	%/h	mA	%/h
1	07:59:20	17.18	0.48	0.84	0.02
2	08:08:23	26.60	0.75	0.83	0.02
3	07:44:16	15.84	0.45	0.87	0.02
4	07:51:32	15.87	0.45	0.81	0.02
5	07:36:18	16.55	0.47	0.84	0.02
**Median**	**07:51:32**	**16.55**	**0.47**	**0.84**	**0.02**

## Data Availability

Data are contained within the article.
